# *Quasipaa spinosa-Derived Parvalbumin* Attenuates Exercise-Induced Fatigue via Calcium Homeostasis and Oxidative Stress Modulation in Exhaustively Trained Mice

**DOI:** 10.3390/nu17122043

**Published:** 2025-06-19

**Authors:** Kai Sang, Congfei Lu, Yangfan Zhang, Qi Chen

**Affiliations:** 1 Fujian Key Laboratory of Innate Immune Biology, Biomedical Research Center of South China, College of Life Sciences, Fujian Normal University, Qishan Campus, Fuzhou 350117, China; 2 School of Physical Education and Sport Science, Fujian Normal University, Fuzhou 350117, China; lucongui19870613@fjnu.edu.cn

**Keywords:** *Quasipaa spinosa*, calcium homeostasis, anti-fatigue, exercise performance, anti-muscle atrophy

## Abstract

**Background:** *Quasipaa spinosa* crude extract (QSce), a natural source rich in proteins such as parvalbumin (PV), has been traditionally used to promote physical recovery. However, its mechanisms in mitigating exercise-induced fatigue remain unclear. **Methods:** Using a murine treadmill exhaustion model, we evaluated the effects of QS-derived Parvalbumin (QsPV) (30 and 150 mg/kg/day) on endurance capacity, oxidative stress, tissue injury, and muscle function. Indicators measured included time to exhaustion, intracellular calcium levels, antioxidant enzymes [superoxide dismutase (SOD), glutathione peroxidase (GSH-Px)], lipid peroxidation (malondialdehyde, MDA), injury markers [creatine kinase (CK), lactate dehydrogenase (LDH), cardiac troponin I (cTnI)], renal function (blood urea), and muscle force. **Results:** QsPV-150 significantly increased time to exhaustion by 34.6% compared to the exercise-only group (*p* < 0.01). It reduced MDA by 41.2% in skeletal muscle and increased SOD and GSH-Px levels by 35.4% and 28.1%, respectively. Serum CK, LDH, and cTnI were reduced by 39.5%, 31.7%, and 26.8%, respectively, indicating protection against muscle and cardiac injury. QsPV also decreased blood urea by 22.3% and improved renal histology, with reduced glomerular damage and tubular lesions. At the molecular level, QsPV restored calcium balance and downregulated calpain-1/2 and atrophy-related genes (MuRF-1, MAFbx-32). Muscle contractile force (GAS and SOL) improved by 12.2–20.3%. **Conclusions:** QsPV attenuates exercise-induced fatigue through multi-organ protection involving calcium buffering, oxidative stress reduction, and anti-atrophy effects. These findings support its potential as a natural recovery-enhancing supplement, pending further clinical and pharmacokinetic studies.

## 1. Introduction

Exercise-induced fatigue is defined as the inability to maintain expected exercise intensity or performance, often accompanied by metabolic and structural stress across multiple organs [1,2,3,4]. In athletes and individuals undergoing high-intensity training, fatigue can lead to tissue injury, immune suppression, and chronic overtraining syndrome, making fatigue prevention and recovery key concerns in sports medicine and public health [5,6].

The underlying mechanisms of fatigue are multifactorial. The rapid depletion of energy substrates such as glucose and glycogen, coupled with the accumulation of metabolites like lactate and urea nitrogen, impairs muscle contractility [7]. Additionally, intense exercise elevates reactive oxygen species (ROS), disrupting redox balance and damaging cellular membranes, mitochondria, and contractile proteins in cardiac and skeletal muscle [8,9,10]. This oxidative stress also contributes to systemic inflammation and organ dysfunction.

Nutritional strategies targeting oxidative damage and energy metabolism have shown promise in alleviating fatigue [11,12,13,14,15,16,17,18]. Compounds such as creatine, polyphenols, and amino acid derivatives can delay exhaustion and reduce exercise-induced injury. However, the efficacy of many of these interventions remains limited in scope or poorly defined mechanistically.

*Quasipaa spinosa* (QS) is a large amphibian species native to southern China and has traditionally been regarded as a functional food with nourishing properties. Previous studies have shown that the protein content in QS muscle reaches 18.55–19.39%, indicating its high nutritional and potential physiological value [19]. Proteins and peptides in QS are believed to contribute to its restorative effects, particularly in the context of physical performance and fatigue recovery. Despite its long history of traditional use, the functional components of QS and their mechanisms of action in exercise-related fatigue remain poorly characterized. Our recent proteomic analysis identified parvalbumin (PV) as the most abundant protein (~23%) in QS-derived crude extract (QSce). PV is a calcium-binding protein known to regulate calcium homeostasis and oxidative balance in muscle tissue, suggesting it may be a key active component contributing to QSce’s anti-fatigue potential.

The present study aimed to investigate the effects of purified QS-derived parvalbumin (QsPV) on fatigue induced by exhaustive exercise. We evaluated physiological outcomes such as endurance capacity, calcium homeostasis, oxidative stress levels, muscle and organ injury markers, and contractile function in a mouse model. In addition, mechanistic insight was sought to elucidate how QsPV mediates its protective effects and facilitates recovery following intense physical stress.

## 2. Materials and Methods

### 2.1. Preparation of Quasipaa spinosa Crude Extract and Placebo

*Quasipaa spinosa* (QS) specimens were provided by Fujian ShiDongWang Ecological Culture Co., Ltd. (Quanzhou, China). Freshly sacrificed QS specimens were decapitated and eviscerated. The muscle tissue was homogenized in 3 volumes of 20 mmol/L Tris-HCl buffer (pH 7.5) using a tissue disruptor. The homogenate was centrifuged at 15,000× *g* for 30 min at 4 °C. The resulting supernatant was collected as the QS crude extract.

### 2.2. Protein Composition Analysis

The total soluble proteins in QSce were profiled using a SWATH-MS proteomics workflow (ZenoTOF 7600, AB Sciex Pte. Ltd., Framingham, MA, USA). Relative abundances were calculated by peak area integration. Parvalbumin constituted approximately 23% of total protein (Table 1 and Table S3).

### 2.3. Purification and Characterization of QsPV

Parvalbumin (QsPV) was isolated from QSce by Hengzheng Biotechnology Co., Ltd. (Fuzhou, China) using ion-exchange chromatography and size exclusion chromatography. Detailed purification steps, including buffers, column types, and elution gradients, are provided in Supplementary Material File S1 to ensure reproducibility. Protein identity and purity were confirmed via LC-MS/MS and SDS-PAGE (>95% purity). Quantification was performed using a BCA assay.

### 2.4. Animal Experimental Design

All animals were housed in accordance with institutional guidelines for laboratory animals, and the experimental protocols were approved by the Fujian Normal University Institutional Animal Care and Use Committee (IACUC Approval No. 2023-0029, approval date: 22 February 2023). A total of 50 male C57BL/6J mice (6-weeks-old) were obtained from Shanghai Slake Laboratory Animal Technology Co., Ltd. (Shanghai, China). The mice were housed in controlled specific pathogen-free conditions under moderate humidity (45–55%), a room temperature of 22 ± 2 °C, and a 12-h light–dark cycle. Mice were provided with a standard diet and had ad libitum access to water. Following a 7-day acclimatization period, mice failing to adapt to treadmill running at 20 m/min were excluded. The remaining 40 mice were randomly allocated into four groups using complete randomization (n = 10/group): Normal control (NC), Exhaustive exercise (E), 30 mg/kg QS crude extract (QSce 30), and 150 mg/kg QS crude extract (QSce 150), or 30 mg/kg QS-derived Parvalbumin (QsPV 30), and 150 mg/kg QS-derived Parvalbumin (QsPV 150) [20]. The exercise program was performed using the following protocol: Running at 15 m/min for 3 min to warm up, and then increasing the speed 2 m/min every minute until 21 m/min. After that, the speed was increased at a rate of 1 m/min every 2 min. Mice were considered exhausted when they refused to run even if they were electrically stimulated [21]. On the last day, all mice rested for 30 min after running and were sacrificed. Supplementation was given orally for 21 days prior to and throughout the exercise protocol. Sample size (n = 10/group) was determined via power analysis (G Power v3.1; effect size = 1.5, α = 0.05, power = 0.8) based on pilot exhaustion time data. 

### 2.5. Experimental Protocol for Isometric Muscle Force Measurement

#### 2.5.1. System Preparation

Activate the murine muscle force testing system by sequentially powering the current controller, data acquisition unit, and force transducer. Configure the system for ex vivo testing mode. Assemble the organ bath, mounting arm, and oxygen supply valve. Initiate the external circulating water bath (28 °C ± 0.5 °C) and connect to the organ bath. Fill the bath with oxygenated (95% O_2_, 5% CO_2_) Krebs–Ringer solution (28 °C), maintaining continuous gas perfusion (≈1–2 bubbles/s) via a calibrated flowmeter.

#### 2.5.2. Muscle Mounting & Stabilization

Suspend the isolated muscle strip between two platinum electrodes: attach one end to the force transducer’s mounting arm and secure the opposite end to the fixed bath hook. Align the muscle parallel to the electrode array. Apply 5 mN preload via micrometer adjustment. Allow 10-min equilibration under resting tension.

#### 2.5.3. Parameter Optimization

Optimal stimulation current determination stimulation protocol: Set 0.4 Hz train frequency (60 s duration). Increment current intensity by 50 mA until maximal isometric force plateau (Fmax). Maintain post-test equilibration for 3 min.

#### 2.5.4. Optimal Resting Length Determination

Stimulation protocol: Use 0.01 Hz single pulses (1 s duration). Stretch muscle in 0.5 mN increments until Fmax stabilization. Record optimal length (Lo) via digital caliper (±0.01 mm). Post-test equilibration: 3 min.

#### 2.5.5. Contractile Property Assessment

Twitch contraction analysis: Deliver three single stimuli (60 s inter-stimulus interval). Record parameters of absolute twitch force, maximal rate of force development (+dF/dtMax), and maximal relaxation rate (−dF/dtMin). Post-test equilibration: 3 min.

#### 2.5.6. Tetanic Contraction Analysis

Perform fused tetanus protocol (frequency-dependent). Terminate experiment, excise muscle for wet weight measurement. Record parameters of absolute tetanic force, +dF/dtMax, and −dF/dtMin.

#### 2.5.7. Data Documentation

Quantitative parameters from twitch and tetanic contractions were systematically tabulated (Table S1).

#### 2.5.8. Relative Muscle Force Calculation

Muscle Cross-Sectional Area (CSA, mm^2^) = Muscle Wet Weight (mg)/Muscle Length (mm) × 1.06 (density constant, g/cm^3^)

Relative Muscle Tension (mN/mm^2^) = Absolute Muscle Force (mN)/CSA (mm^2^)

### 2.6. Sample Collection and Processing

Mice were anesthetized with 5% sodium pentobarbital immediately post-exhaustion. Blood samples were collected via enucleation prior to euthanasia by cervical dislocation. The gastrocnemius and myocardium were rapidly dissected, rinsed in ice-cold saline (4 °C), and fixed in 4% paraformaldehyde for paraffin sectioning. Whole blood was allowed to clot at room temperature for 30 min, centrifuged (3000× *g* rpm, 15 min), and serum aliquots were stored at −80 °C for ELISA.

### 2.7. Skeletal Muscle Calcium Content Determination

Minced tissues were homogenized in ice-cold lysis buffer (10 mM Tris-HCl pH 7.4, 1% Triton X-100, protease inhibitor cocktail [Sigma, St. Louis, MO, USA, complete™]). After centrifugation (14,000× *g*, 5 min, 4 °C), supernatants were diluted 1:5 in assay buffer. Calcium content was determined using a colorimetric kit (Byotime, Wuhan, China, S1063S) following incubation at 37 °C for 20 min. Absorbance was measured at 575 nm (SpectraMax M5, Molecular Devices (Shanghai) Co., Ltd., Shanghai, China), with standards (0–100 μg/mL) run in parallel. Calcium content calculation: C = (A × n)/V (μg/μL).

C: Calcium concentration in muscle homogenate; A: Calcium content (μg) determined from standard curve; n: Dilution factor; V: Sample volume (μL) added.

### 2.8. Enzyme Linked Immunosorbent Assay (ELISA)

Mouse blood samples were allowed to clot at room temperature for 30 min, and serum separation was achieved through centrifugation at 3000 rpm for 15 min. Sample dilutions were 1:10 for serum-based ELISA (CK, LDH, cTnI, etc.) and 1:20 for tissue extracts. Incubations were carried out at 37 °C for 30–60 min depending on the assay type. The expression levels of creatine kinase (CK), blood lactate (LDH), cardiac troponin I (cTn I), myoglobin, superoxide dismutase (SOD), malondialdehyde (MDA), and glutathione peroxidase (GSH-PX) in both supernatant and serum samples were assessed using ELISA kits (Sandwich) (MEIKEBIO, Suzhou, China; CK: MK5951A, MK0863A; LDH: MK5900A;cTn I: MK3163B; Myoglobin: MK0921A. Nanjing Jiancheng Bioengineering Institute, Nanjing, China, SOD: A001-1-1;MDA: A003-1-2; GSH-PX: A005-1-1) in accordance with the manufacturer’s instructions.

### 2.9. Histopathological Examination and Histological Scoring

The histopathological examinations of kidney, gastrocnemius, and cardiac tissues—including sectioning, staining, and analysis—was performed by Wuhan Servicebio Technology Co., Ltd. (Wuhan, China), with fibrosis quantification via collagen-positive area measurement using Masson’s trichrome staining and ImageJ (Version: 1.54k) analysis [22]; kidney injury assessment [23] employed a semi-quantitative scoring system for tubular degeneration, interstitial edema, and glomerular damage as previously reported through the use of 2.10. RNA Extraction and Quantitative Real-Time PCR (qRT-PCR).

The total RNA extraction, reverse transcription, and qRT-PCR process were performed as described previously [24]. Primer sequences are presented in Table S2. GAPDH and 28S were used as the reference genes in the liver and ileum, respectively.

### 2.10. Statistical Analysis

Images were processed using Illustrator 2020 (Adobe, San Jose, CA, USA). All data are expressed as mean values ± SEM. Unpaired two-tailed t-tests and one-way analyses of variance (ANOVA) were performed to compare groups, followed by Tukey’s post hoc tests. All statistical analyses were carried out using GraphPad Prism 8 (v8.0; GraphPad Software, San Diego, CA, USA). *p* < 0.05 was considered statistically significant.

## 3. Results

### 3.1. QS Crude Extract Enhances Exercise Performance in Mice

**Figure 1:**
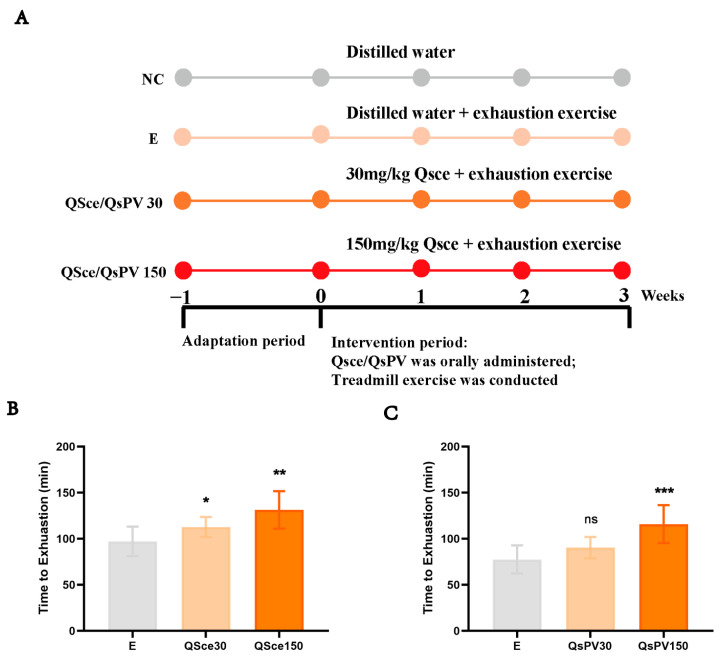
Effect of QS crude extract on time to exhaustion in mice. (**A**) The exhaustion exercise protocol, with mice receiving QSce (30 or 150 mg/kg/d) or QsPV (30 and 150 mg/kg/d). (**B**,**C**) Time to exhaustion in mice undergoing the 21-day exercise exhaustion protocol with 30 or 150 (mg/kg/d) dose QSce and QsPV. All values are expressed as means ± SEM. (n = 8. * *p* < 0.05, ** *p* < 0.01, *** *p* < 0.001).

To evaluate the anti-fatigue potential of *Quasipaa spinosa* products, mice were subjected to a standardized exhaustive treadmill protocol. Compared to the E (exhaustive exercise only) group, both the QS crude extract (QSce)-30 and the QSce-150 group exhibited significantly longer running durations before exhaustion, with the higher dose group showing a greater effect (Figure 1B). This observation affirmed that QSce supplementation contributed to delaying the onset of exercise fatigue and augmenting the mice’s athletic capabilities.

### 3.2. Proteomic Profiling Identifies Parvalbumin as the Dominant Protein in QSce

The proteomic analysis of QSce identified a total of 10 major soluble proteins. Parvalbumin accounted for approximately 23.1% of total protein content (Table 1), followed by creatine kinase M-type and collagen alpha-1(I) chain. The full protein profile is provided in Supplementary Table S3. This high abundance suggests that PV may play a key role in mediating QSce’s physiological effects.

### 3.3. QS-Derived Parvalbumin Alleviated Intense Exercise-Induced Fatigue and Oxidative Stress Response

First, we repeated the exhaustive exercise protocol in mice, and the results demonstrated that the supplementation of QS-derived Parvalbumin (QsPV) alone could prolong the time to exhaustion, although no statistically significant difference in exhaustion time was observed between the QsPV30 group and the E group (Figure 1C).

**Figure 2:**
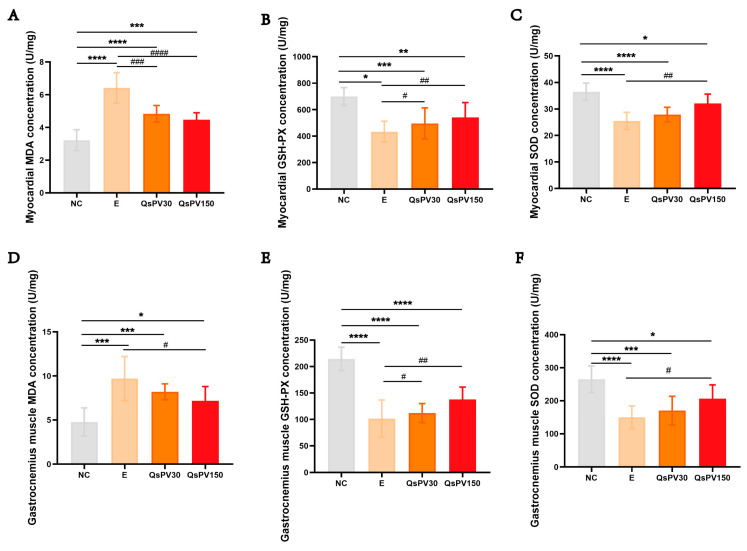
Levels of oxidative stress and anti-oxidant markers in myocardial muscle and gastrocnemius muscle from mice after the three-week exercise exhaustion protocol. Levels of oxidative stress and anti-oxidant markers in (**A**–**C**) myocardial muscle and (**D**–**F**) gastrocnemius muscle from mice after the three-week exercise exhaustion protocol. All values are expressed as means ± SEM. (* *p* < 0.05, ** *p* < 0.001, *** *p* < 0.005, **** *p* < 0.0001, NC vs. E and QsPVs; ^#^ *p* < 0.05, ^##^ *p* < 0.01, ^###^ *p* < 0.005, ^####^ *p* < 0.0001, E vs. QsPVs).

Exhaustive treadmill running led to significant oxidative stress in both skeletal and cardiac muscles, as shown by increased malondialdehyde (MDA) levels and decreased antioxidant enzyme activities (SOD and GSH-Px) in the E group compared to NC. QsPV supplementation dose-dependently reversed these changes: QsPV-150 reduced MDA levels by 41.2% and increased SOD and GSH-Px activities by 35.4% and 28.1%, respectively, in both muscle types (Figure 2A–F).

**Figure 3:**
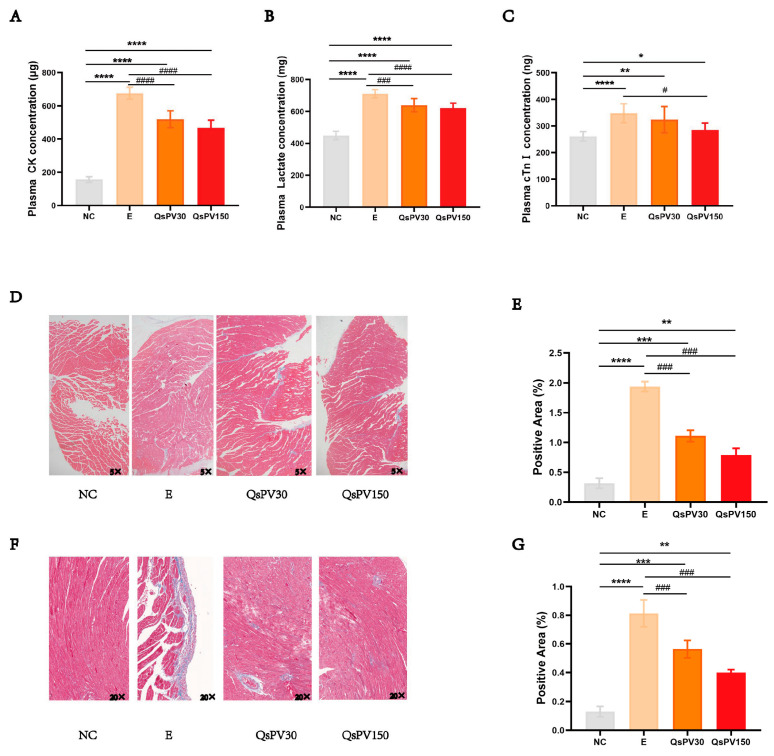
Serum injury biomarkers and fibrotic outcomes in skeletal muscle and myocardium. (**A**–**C**) Serum levels of injury biomarkers in mice after 3-week training: (**A**) Creatine kinase (CK), (**B**) Lactate dehydrogenase (LDH), (**C**) Cardiac troponin I (cTnI). (**D**–**G**) Masson’s trichrome staining of gastrocnemius and myocardial tissues in mice after 3-week training: (**D**) Representative micrographs of gastrocnemius Masson staining, (**E**) Percentage of positively stained area in gastrocnemius, (**F**) Representative micrographs of cardiac Masson staining, (**G**) Percentage of positively stained area in cardiac tissue. All values are expressed as means ± SEM. (* *p* < 0.05, ** *p* < 0.001, *** *p* < 0.005, **** *p* < 0.0001, NC vs. E and QsPVs; ^#^ *p* < 0.05, ^###^ *p* < 0.001, ^####^ *p* < 0.0001, E vs. QsPVs. n = 8).

We also assessed tissue injury markers in serum. Creatine kinase (CK), lactate dehydrogenase (LDH), and cardiac troponin I (cTnI) levels were significantly elevated in E mice, indicating skeletal and myocardial damage. QsPV-150 significantly reduced CK by 39.5%, LDH by 31.7%, and cTnI by 26.8% compared to E (Figure 3A–C), while QsPV-30 showed partial but significant reductions.

Histological assessment by Masson’s trichrome staining revealed increased collagen deposition in both gastrocnemius and myocardial tissues in the E group, indicative of early fibrotic remodeling. QsPV treatment reduced collagen-positive area by approximately 30–40% (*p* < 0.05), with more pronounced effects at the higher dose (Figure 3D–G). Quantification of staining confirmed the protective effect of QsPV against structural damage and fibrotic response following intense physical stress.

### 3.4. QsPV Supplementation Reduces Renal Impairment in Mice

**Figure 4:**
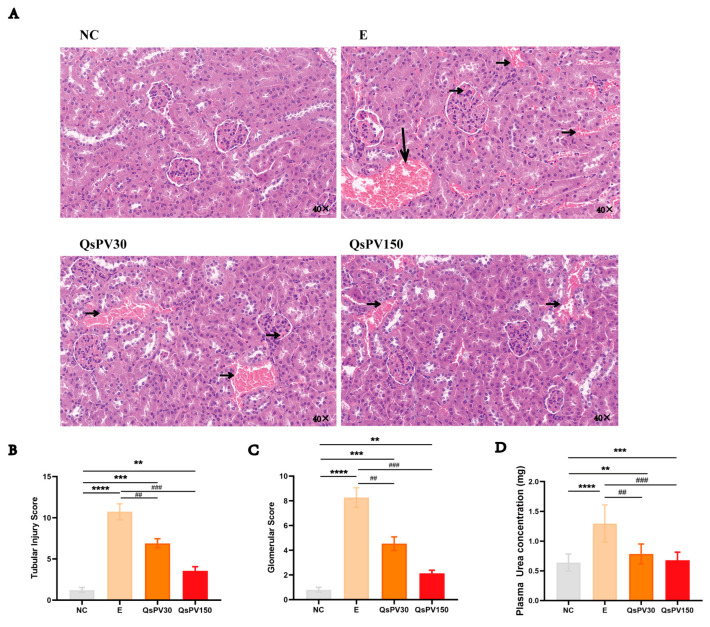
Renal injury with exhaustive exercise is recovered with QsPVs supplementation. (**A**) Pathological examination of HE-stained mouse kidney sections (5×). Arrows indicate glomerular and tubular lesions. (**B**) Renal tubular score. (**C**) Glomerular score. (**D**) Concentration of urea in mouse peripheral blood plasma (mg) in mice. All values are expressed as means ± SEM. (** *p* < 0.001, *** *p* < 0.005, **** *p* < 0.0001, NC vs. E and QsPVs; ^##^ *p* < 0.05, ^###^ *p* < 0.005, E vs. QsPVs, n = 8).

Exhaustive exercise led to significant renal dysfunction, as evidenced by elevated plasma urea levels in the E group compared to NC (Figure 4B). QsPV supplementation significantly reduced blood urea concentrations in both QsPV30 and QsPV150 groups, with a clear dose-response trend.

Histopathological analysis (H&E staining) revealed glomerular congestion, vascular bruising, and tubular epithelial damage in E mice (Figure 4A–C). In contrast, kidneys from QsPV-treated groups showed notably reduced structural abnormalities, with less glomerular swelling and preserved capillary integrity. While minor lesions were still observed, lesion severity and frequency were significantly lower than in E. These findings demonstrate the beneficial impact of QsPV supplementation in protecting against renal injuries in mice subjected to exhaustive exercise.

### 3.5. QsPV Restores Calcium Homeostasis in Skeletal Muscle

Calcium dysregulation is a hallmark of muscle fatigue. Compared to NC, E mice exhibited significantly elevated intracellular calcium concentrations in both soleus (SOL) and gastrocnemius (GAS) muscles (Table 2). QsPV150 supplementation reduced Ca^2+^ content by 43.2% in SOL and 27.2% in GAS compared to E, restoring levels close to baseline. This suggests that QsPV contributes to preserving excitation–contraction coupling by buffering intracellular calcium under fatigue conditions.

### 3.6. QsPV Suppresses Muscle Atrophy-Related Gene Expression

**Figure 5:**
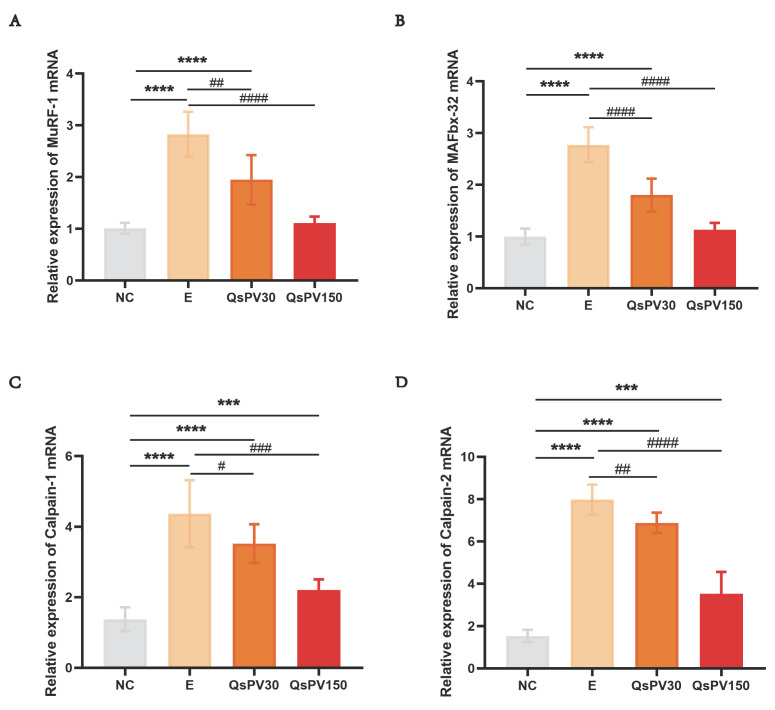
Expression analysis of genes associated with skeletal muscle atrophy in mice. (**A**,**B**) MAFbx-32 and MuRF-1 mRNA expression in skeletal muscle; (**C**,**D**) Calpain1/2 mRNA expression in skeletal muscle. All values are expressed as means ± SEM. (*** *p* < 0.005, **** *p* < 0.0001, NC vs. E and QsPVs; ^#^ *p* < 0.01, ^##^ *p* <0.05, ^###^ *p* < 0.001, ^####^ *p* <0.0001, E vs. QsPVs, n = 8).

To assess molecular markers of exercise-induced muscle degradation, we examined the expression of muscle-specific atrogenes (MAFbx-32, MuRF-1) and calcium-activated proteases (Calpain-1, Calpain-2) in skeletal muscle. In the E group, all four genes were significantly upregulated compared to NC (Figure 5A–D). QsPV30 and QsPV150 supplementation downregulated the expression of MAFbx-32 and MuRF-1 by 22–45% and reduced Calpain-1/2 expression by 30–40% compared to E (*p* < 0.05). These findings suggest that QsPV mitigates proteolytic signaling and muscle atrophy via calcium buffering and transcriptional modulation.

### 3.7. QsPV Improves Tetanic Muscle Contractile Function

The contractile function of SOL and GAS muscles was assessed by ex vivo tetanic stimulation. Compared to NC, E mice showed 31–44% reductions in both absolute and relative muscle force (Table 3). QsPV supplementation partially reversed this decline: QsPV-30 improved relative force by 28.5% in SOL and 34.7% in GAS (*p* < 0.05 vs. E). Similarly, maximal rates of contraction and relaxation (±dF/dt) were impaired in E muscles (Table 4 and Table 5). QsPV treatment significantly increased +dF/dt and −dF/dt values, indicating improved contractile dynamics.

### 3.8. QsPV Enhances Contractile Kinetics of Fatigued Muscle

In addition to restoring peak tetanic force, we further examined contractile velocity characteristics by measuring the maximal rates of force development (+dF/dt) and relaxation (−dF/dt) during tetanic stimulation. Compared to the NC group, +dF/dt was significantly reduced by 44.1% in soleus (SOL) muscles and 25.3% in gastrocnemius (GAS) muscles in the E group, indicating impaired contractile kinetics (Table 4). QsPV supplementation improved +dF/dt by 14.0% in SOL and 7.4% in GAS compared to E. Similarly, −dF/dt, representing the maximal rate of relaxation, was reduced by 43.3% in SOL and 39.4% in GAS in E mice (Table 5). QsPV treatment significantly improved these values, with the QsPV group showing a 28.0% increase in SOL and a 37.3% increase in GAS compared to E (*p* < 0.05). These improvements in contractile dynamics suggest that QsPV not only enhances absolute force production but also accelerates excitation–contraction and relaxation coupling kinetics, likely through improved calcium handling and reduced muscle damage.

## 4. Discussion

This study reveals that *Quasipaa spinosa*-derived parvalbumin (QsPV) exerts multifaceted protective effects against exercise-induced fatigue, including improvements in endurance, calcium homeostasis, oxidative stress, tissue integrity, and muscle contractility. The systemic benefits observed in skeletal muscle, myocardium, and kidney function suggest that QsPV acts not merely as a local modulator but as a systemic homeostatic regulator under physical stress conditions.

Parvalbumin is a cytosolic calcium-binding protein highly enriched in fast-twitch muscle fibers, known for its rapid Ca^2+^ sequestration following contraction [24,25,26]. During exhaustive exercise, sustained depolarization and ATP depletion impair calcium reuptake by the sarcoplasmic reticulum, resulting in intracellular Ca^2+^ accumulation [27]. This overload activates calcium-dependent proteases such as calpain-1 and calpain-2, which degrade structural and regulatory muscle proteins, leading to contractile dysfunction and tissue injury [28]. QsPV supplementation restored calcium levels and suppressed calpain expression, suggesting that it functions as a molecular “calcium sink,” thereby breaking the calcium overload–injury cycle.

In addition to protease activation, excessive intracellular Ca^2+^ disrupts mitochondrial homeostasis, triggering the opening of permeability transition pores and enhancing electron leakage in the respiratory chain, which boosts reactive oxygen species (ROS) production [29,30,31]. Elevated ROS initiates lipid peroxidation, protein oxidation, and organelle damage, which are central to oxidative stress-mediated fatigue and muscle degeneration [32,33]. Our findings showed that QsPV significantly reduced MDA levels and restored the activities of key antioxidant enzymes (SOD, GSH-Px), particularly in muscle and cardiac tissues. These effects suggest that QsPV indirectly restores mitochondrial redox balance by preventing Ca^2+^-induced mitochondrial stress.

ROS also plays a signaling role in regulating skeletal muscle catabolism [21,32]. Previous studies have shown that oxidative stress activates redox-sensitive transcriptional pathways, leading to the upregulation of E3 ubiquitin ligases such as MuRF-1 and MAFbx-32, which promote the proteasomal degradation of contractile proteins [25,26,34,35]. In our study, QsPV significantly downregulated MuRF-1 and MAFbx-32 expression in skeletal muscle, especially at the higher dosage. These findings suggest that QsPV may preserve muscle mass by suppressing oxidative stress-induced catabolic signaling.

**Figure 6:**
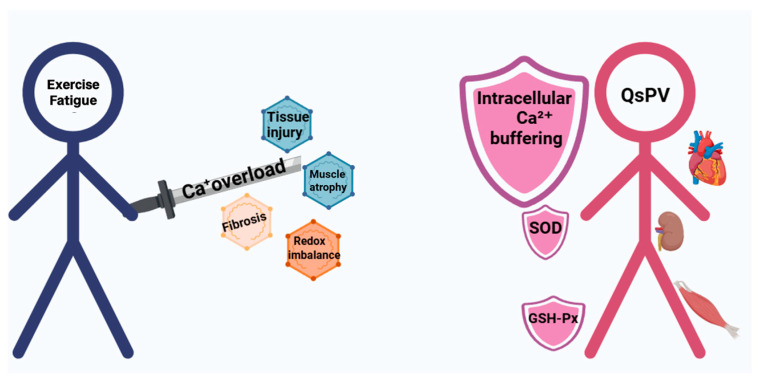
Proposed mechanistic model of QsPV in alleviating exercise-induced fatigue.

Together, these results support a coherent mechanistic cascade in which QsPV buffers intracellular calcium, thereby stabilizing mitochondrial function, reducing reactive oxygen species (ROS) production, downregulating the expression of MuRF-1 and MAFbx-32, and ultimately preserving muscle structure and function. This cascade aligns with the observed improvements in endurance time, muscle contractility (+dF/dt, −dF/dt), and reductions in injury markers (CK, LDH, cTnI, and blood urea), as summarized in Figure 6.

Beyond skeletal muscle, QsPV also demonstrated protective effects in the heart and kidney. In cardiac tissue, exercise-induced damage involves ROS accumulation, calcium dysregulation, and inflammatory remodeling, which lead to fibrosis and contractile impairment [36,37,38]. QsPV reduced myocardial collagen deposition and preserved histological structure, suggesting anti-fibrotic and antioxidant effects. Similarly, the kidneys, which are sensitive to exercise-induced hypoperfusion and oxidative injury, showed improved histopathology and lower urea levels after QsPV treatment. While the precise mechanisms remain to be clarified, these effects may result from improved systemic redox balance and cellular stress resistance.

Dose-dependent effects of QsPV were observed in several key parameters, including endurance time, antioxidant capacity, calcium homeostasis, and atrogene expression. While both 30 mg/kg and 150 mg/kg doses produced beneficial outcomes, the higher dose consistently resulted in greater improvements. The dosage selection in this study was guided by previous literature and pre-experimental testing, which demonstrated good tolerability without adverse effects. Further studies are warranted to establish a formal dose–response relationship and long-term safety profile. Importantly, although our results indicate that QsPV reproduces the key anti-fatigue effects of the crude extract (QSce), additional investigations are needed to fully confirm its role as the primary active component. Our proteomic analysis identified PV as the most abundant protein in QSce (~23%), and the observed biological outcomes of purified QsPV support its dominant contribution. However, we acknowledge the potential for synergistic interactions with other minor proteins or peptides. Future studies will aim to perform direct functional comparisons between QsPV and other extract components, as well as employ knockdown or inhibition models to definitively validate its mechanistic role.

Moreover, the current findings are limited to the acute post-exercise phase. Although QsPV improved immediate muscle function and biochemical markers, we did not assess delayed recovery time points. Evaluating muscle force restoration or metabolic profiles over extended recovery periods (e.g., 3–7 days post-exercise) will be crucial to determine the sustained efficacy of QsPV and its potential relevance for rehabilitation or chronic fatigue management.

In the context of translational applications, we have briefly discussed the potential relevance of QsPV in sports nutrition and functional recovery. Compared to existing natural or commercial anti-fatigue agents—such as ginseng, taurine, or marine peptides—QsPV offers a distinctive mechanism based on calcium buffering and mitochondrial protection. Its protein-based nature may also facilitate formulation into targeted dietary supplements. However, further comparisons of efficacy, bioavailability, and long-term safety will be essential for practical development.

Despite these promising findings, several limitations should be acknowledged. First, the long-term effects of QsPV following treatment cessation were not evaluated, and no pharmacokinetic or bioavailability studies were conducted. Second, although a QsPV-only group (without exercise) was monitored for body weight and food intake (Table S7), its physiological effects under baseline conditions remain unclear. Third, while two doses were tested, a comprehensive dose–response analysis and toxicity evaluation are still needed to inform future applications. In addition, follow-up beyond the immediate post-exercise period was not performed; future studies should assess delayed recovery to determine the sustained efficacy of QsPV over time.

Exhaustive exercise leads to calcium overload, mitochondrial dysfunction, and increased reactive oxygen species (ROS) production. These stress responses promote the activation of calcium-dependent proteases (calpain-1/2) and the upregulation of muscle atrophy-related genes (MuRF-1, MAFbx-32), resulting in impaired contractility and tissue damage. QsPV buffers intracellular Ca^2+^, thereby stabilizing mitochondrial function and reducing ROS generation. This cascade attenuates oxidative stress and suppresses proteolytic and atrophic signaling, ultimately preserving muscle structure and improving systemic endurance. The protective effects of QsPV extend beyond skeletal muscle to cardiac and renal tissues, indicating a multi-organ protective mechanism against exercise-induced fatigue.

## 5. Conclusions

This study demonstrates that *Quasipaa spinosa*-derived parvalbumin (QsPV) alleviates exercise-induced fatigue through a multi-target mechanism involving calcium buffering, oxidative stress mitigation, and the suppression of muscle atrophy signaling. QsPV supplementation improved endurance, preserved muscle contractility, reduced biochemical and histological markers of tissue injury, and provided protection to the skeletal muscles, heart, and kidney following exhaustive exercise. Mechanistically, our findings support a sequential protective cascade: Ca^2+^ homeostasis→mitochondrial stabilization→ROS reduction→downregulation of MuRF-1/MAFbx-32, resulting in improved muscle function and systemic recovery. This integrated pathway highlights QsPV as the key functional component of *Quasipaa spinosa* extract. While these results suggest the potential of QsPV as a naturally derived recovery-enhancing compound, further studies are warranted to explore its pharmacokinetics, chronic safety, and efficacy across sexes and long-term fatigue models. Clinical translation will also require investigation in human or large-animal systems.

## Figures and Tables

**Table 1:** Major soluble proteins identified in *Quasipaa spinosa* crude extract (QSce) by LC-MS/MS ^1^.

Protein Name	Contents (%)
parvalbumin	23.075062372
creatine kinase M-type	6.769943806
collagen alpha-1(I) chain	5.526668642
beta-enolase isoform X1	5.450013635
myosin regulatory light chain 2, skeletal muscle isoform-like, partial	4.867337207
fructose-bisphosphate aldolase A	3.576699575
adenylate kinase isoenzyme 1 isoform X2	3.149677331
triosephosphate isomerase	3.084792939
collagen alpha-2(I) chain	2.592852484
alpha-enolase	2.324886664

^1^ Data are presented as percentages of total soluble protein.

**Table 2:** Skeletal muscle Ca^2+^ content(μg/μL).

Group	SOL	GAS
NC	0.083 ± 0.026	0.071 ± 0.005
E	0.162 ± 0.016 **	0.224 ± 0.03 **
QsPV30	0.113 ± 0.022 ^#^	0.142 ± 0.04 ^#^
QsPV150	0.092 ± 0.006 ^##^	0.091 ± 0.014 ^##^

** *p* < 0.001, NC vs. E and QsPVs; *^#^ p* < 0.05, *^##^ p* < 0.01, E vs. QsPVs, n = 8.

**Table 3:** Skeletal muscle tetanic force.

Group	Absolute Muscle Force		Relative Muscle Force	
	SOL	GAS	SOL	GAS
NC	241.36 ± 3.68	358.47 ± 4.35	386.30 ± 2.32	458.94 ± 7.46
E	146.05 ± 3.57 **	253.99 ± 1.73 **	197.32 ± 9.46 **	317.60 ± 9.36 **
E + QsPV (0.6 mg/mL) ^1^	221.39 ± 7.45	301.94 ± 2.31 *	317.25 ± 4.28 *	377.16 ± 10.42 *

^1^ Convert the supplementary dosage to concentration for QsPV150. Formula: ex vivo concentration = Dosage × Tissue mass proportion/Solvent volume. * *p* < 0.01, ** *p* < 0.05, indicates comparison with Group NC.

**Table 4:** Skeletal muscle tetanic contraction Max dF/dt(mN/s).

Group	SOL	GAS
NC	4099.08 ± 160.91	13,628.93 ± 339.04
E	2993.56 ± 179.57 **	9981.77 ± 361.39 **
E + QsPV (0.6 mg/mL)	3505.14 ± 164.64 *	11,444.49 ± 206.16 *

* *p* < 0.01, ** *p* < 0.05, indicates comparison with Group NC.

**Table 5:** Skeletal muscle tetanic contraction Min dF/dt(mN/s).

Group	SOL	GAS
NC	4319.48 ± 260.91	17,078.93 ± 632.90
E	2447.76 ± 179.92 **	10,481.07 ± 961.92 **
E + QsPV (0.6 mg/mL)	3905.14 ± 164.64 *	15,034.49 ± 716.62 *

* *p* < 0.01, ** *p* < 0.05, indicates comparison with Group NC.

## Data Availability

All data presented in this study are available on request from the corresponding authors. The data are not to be uploaded to a publicly accessible database.

## Supplementary Materials

The following supporting information can be downloaded at: https://www.mdpi.com/article/10.3390/nu17122043/s1, Table S1: Quantitative parameters from twitch and tetanic contractions are systematically tabulated; Table S2: Sequence of primers used for the qRT-PCR assays; Table S3: Soluble Proteins per 100 g of QS Crude Extract; Table S4: Skeletal muscle single twitch force; Table S5: Skeletal muscle single twitch contraction Max dF/dt(mN/s); Table S6: Skeletal muscle single twitch contraction Min dF/dt(mN/s), Table S7 Food intake and body weight. Supplementary Material File S1: Purification and identification of PV from *Quasipaa spinosa.*

## Abbreviations

The following abbreviations are used in this manuscript:

ANOVA	analysis of variance
BUN	blood urea nitrogen
CK	creatine kinase
cTn I	cardiac troponin I
ELISA	enzyme-linked immunosorbent assay
GSH-PX	glutathione peroxidase
LDH	blood lactate
MDA	malondialdehyde
QS	*Quasipaa spinosa*
QSce	QS-derived crude extract
QsPV	QS-derived parvalbumin
WT	wild-type
SOD	superoxide dismutase
