# Supplementary material for: Quasipaa spinosa-Derived Parvalbumin Attenuates Exercise-Induced Fatigue via Calcium Homeostasis and Oxidative Stress Modulation in Exhaustively Trained Mice
Source: Nutrients. 2025 Jun 19;17(12):2043. doi: 10.3390/nu17122043 (PMC12196480; doi:10.3390/nu17122043)
Supplement: Supplementary file 1 [file nutrients-17-02043-s001.zip › Supplementary Material S1.pdf]

Purification and identification of PV from *Quasipaa spinosa*.

Freshly sacrificed *Quasipaa spinosa* specimens were decapitated and eviscerated. The muscle tissue was homogenized in 3 volumes of 20 mmol/L Tris-HCl buffer (pH 7.5) using a tissue disruptor. The homogenate was centrifuged at 15,000 × g for 30 min at 4°C. The resulting supernatant was collected as the QS crude extract.

This crude extract was heated at 100°C for 20 min and centrifuged to obtain the heat-soluble PV extract. The PV extract was then subjected to ammonium sulfate precipitation at 60–100% saturation. The precipitate was dissolved in 20 mmol/L Tris-HCl (pH 7.5), dialyzed against the same buffer, and loaded onto a DEAE-Sepharose anion-exchange column. Unbound fractions were collected by elution with 20 mmol/L Tris-HCl (pH 7.5) at a flow rate of 0.6 mL/min. Protein concentration was determined by BCA assay prior to dialysis. SDS-PAGE used 12% resolving gels stained with Coomassie Blue R-250.

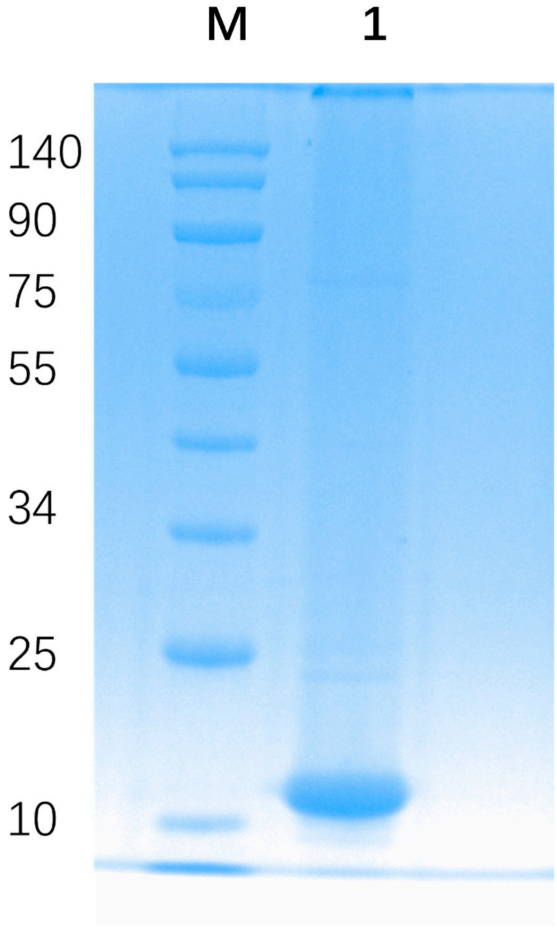

M: protein maker; 1: The final purified PV
