# Supplementary material for: Quasipaa spinosa-Derived Parvalbumin Attenuates Exercise-Induced Fatigue via Calcium Homeostasis and Oxidative Stress Modulation in Exhaustively Trained Mice
Source: Nutrients. 2025 Jun 19;17(12):2043. doi: 10.3390/nu17122043 (PMC12196480; doi:10.3390/nu17122043)
Supplement: Supplementary file 1 [file nutrients-17-02043-s001.zip › Table S1-7.pdf]

Table S1 Quantitative parameters from twitch and tetanic contractions are systematically tabulated

Parameter Settings		SOL	GAS
Single Twitch Contraction	Pulse Width (ms)	0.2	0.2
	Stimulation Frequency (Hz)	1	1
Tetanic Contraction	Pulse Width (ms)	0.2	0.2
	Stimulation Frequency (Hz)	200	200
	Stimulation Duration (ms)	500	500

Table S2 Sequence of primers used for the qRT-PCR assays

Genes	Forward primer (5'-3')	Reverse primer (3'-5')
GAPDH	GTGTTCCTACCCCCAATGTGT-	ATTGTCATACCAGGAAATGAGCTT-
MuRF-1	GCAAACACTGCCACAT	CTTGAGGGGAAAGTGAG
MAFbx-32	AGGACTCCTGCAGAGT	TTCTCGTCCAGGATGGCG
Calpain-1	GCTACCGTTTGTCTA	TAACTCCTCTGTCATCCT
Calpain-2	GGCTTCGGCATCTATGAGG	GAAATCGCCATTCTTG

Table S3. Soluble Proteins per 100 g of QS Crude Extract

Protein name	Contents（%）
Parvalbumin	23.075062372
creatine kinase M-type	6.769943806
collagen alpha-1(I) chain	5.526668642
beta-enolase isoform X1	5.450013635
myosin regulatory light chain 2, skeletal muscle isoform-like, partial	4.867337207
fructose-bisphosphate aldolase A	3.576699575
adenylate kinase isoenzyme 1 isoform X2	3.149677331
triosephosphate isomerase	3.084792939
collagen alpha-2(I) chain	2.592852484
alpha-enolase	2.324886664
troponin C, skeletal muscle isoform X1	2.263270260
phosphoglucomutase-1 isoform X4	2.258198802
L-lactate dehydrogenase A chain	2.235143851
titin	2.156684263
troponin I, fast skeletal muscle	2.085913336
myosin-4-like	1.501834954
serum albumin-like	1.477608345
pyruvate kinase PKM isoform X2	1.147994380
glyceraldehyde-3-phosphate dehydrogenase, partial	1.066290460
collagen alpha-1(II) chain	1.059191909
histone H2B-like	1.056007447

olfactory receptor 497-like	1.046366474
tropomyosin alpha-1 chain isoform X3	0.990077092
alpha-2-macroglobulin-like protein 1, partial	0.984153004
malate dehydrogenase, mitochondrial isoform X1	0.778393778
gamma-crystallin B-like	0.750760316
mimecan	0.719785451
aggrecan core protein	0.681542673
acidic mammalian chitinase-like	0.672163412
aspartate aminotransferase, cytoplasmic	0.633453190
lumican	0.534512361
keratin, type I cytoskeletal 17-like isoform X4	0.533587529
vitellogenin-A2-like	0.504486615

Table S4 Skeletal muscle single twitch force

	Absolute Muscle Force		Relative Muscle Force	
Group	SOL	GAS	SOL	GAS
NC	21.36±1.68	50.47±3.45	26.30±1.32	58.94±4.46
E	20.05±0.57	50.99±2.73	27.32±1.46	57.60±3.36
E+QsPV(0.6mg/ml)	21.39±0.45	51.94±2.38	27.25±1.28	57.16±1.42

Table S5 Skeletal muscle single twitch contraction Max dF/dt(mN/s)

Group	SOL	GAS
NC	1009±70	3428.93±213.04
E	993±50.57	3581±177.39
E+QsPV(0.6mg/ml)	1015±64.64	3444.49±146.16

Table S6 Skeletal muscle single twitch contraction Min dF/dt(mN/s)

Group	SOL	GAS
NC	575.4±43.15	2843.93±113.04
E	583±20.57	2781.00±177.39
E+QsPV(0.6mg/ml)	588±44.64	2841.49±146.16

Table S7 Food intake and body weight

	NC (n=8)	QsPV30(n=8)	QsPV150(n=8)
Body mass (g)	28.64±0.53	28.79±0.67	28.82±0.49
Cardiac mass (mg)	167±2	171±2	161±3
Gastrocnemius-soleus complex mass (mg)	281±3	284±5	281±5
Daily food consumption (g·day^{-1})	3.4±0.36	3.3±1.6	3.63±1.3
